# Supplementary material for: Anatomy and evolution of the first Coleoidea in the Carboniferous
Source: Commun Biol. 2019 Jul 31;2:280. doi: 10.1038/s42003-019-0523-2 (PMC6668408; doi:10.1038/s42003-019-0523-2)
Supplement: Supplementary file 2 — Supplementary Information [file 42003_2019_523_MOESM2_ESM.pdf]

Supplementary Material

Supplementary figures

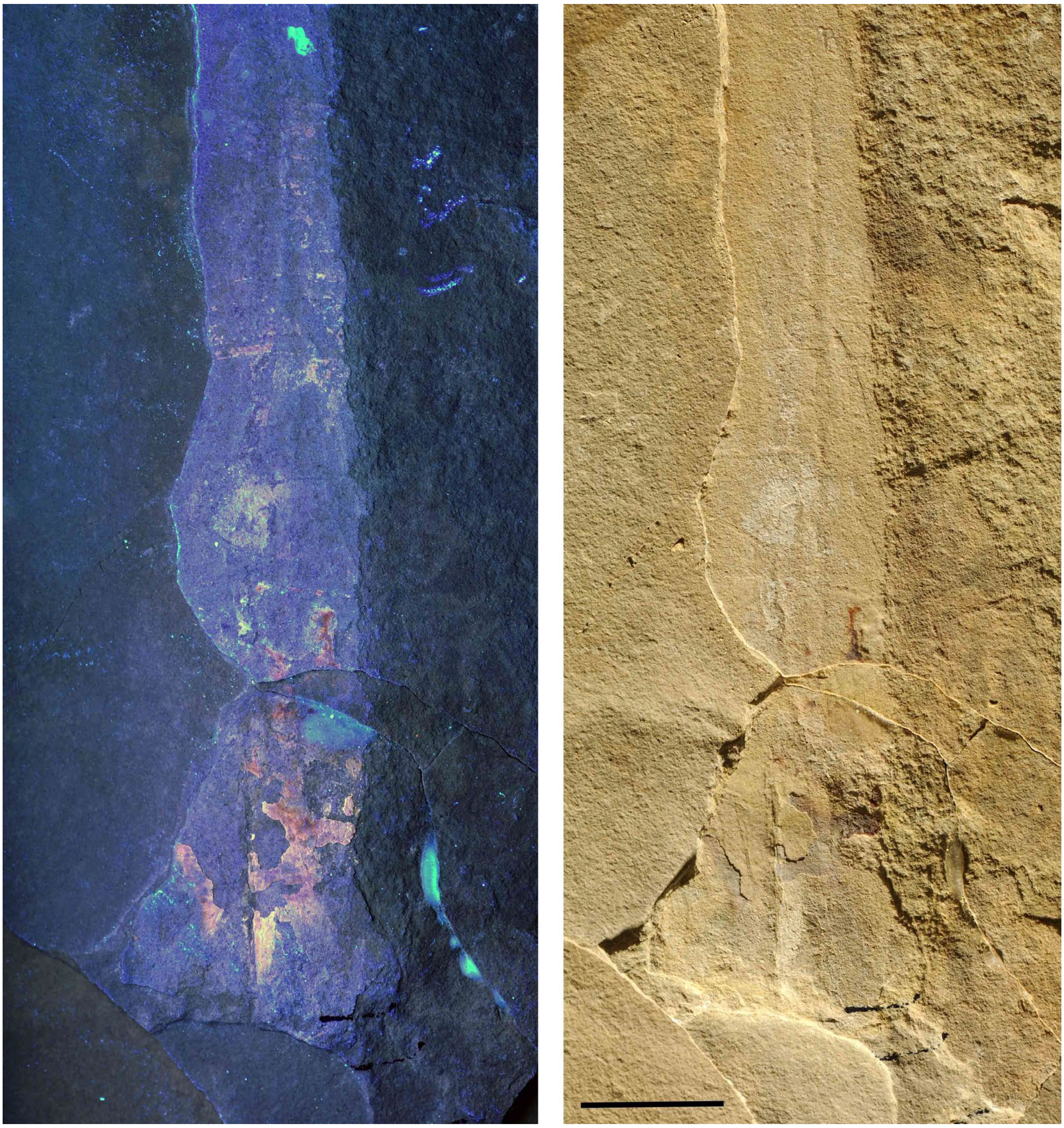

Supplementary Figure 1. *Gordoniconus beargulchensis*, CM 52637. Scale bar is 10 mm. This specimen preserves mainly shell remains and possibly the pellicle (the parts in yellow and orange in A and whitish in B).

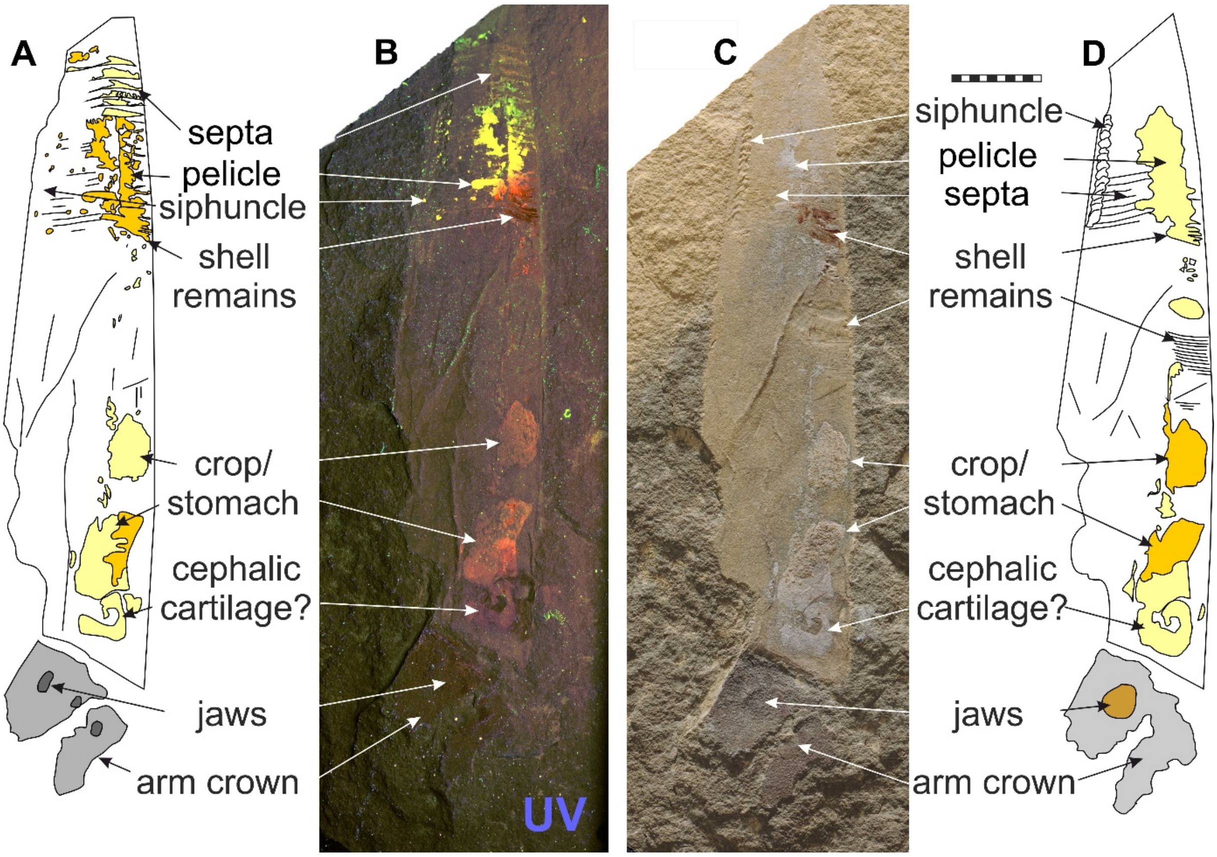

Supplementary Figure 2. *Gordoniconus beargulchensis*, CM 52658. Scale bar is 10 mm. This is the second specimen except the holotype showing arm crown remains. It is seen in lateral aspect with the venter on the left as indicated by the siphuncle. A, sketch of B. B, UV-image. C, photo taken under white light. D, sketch of C.

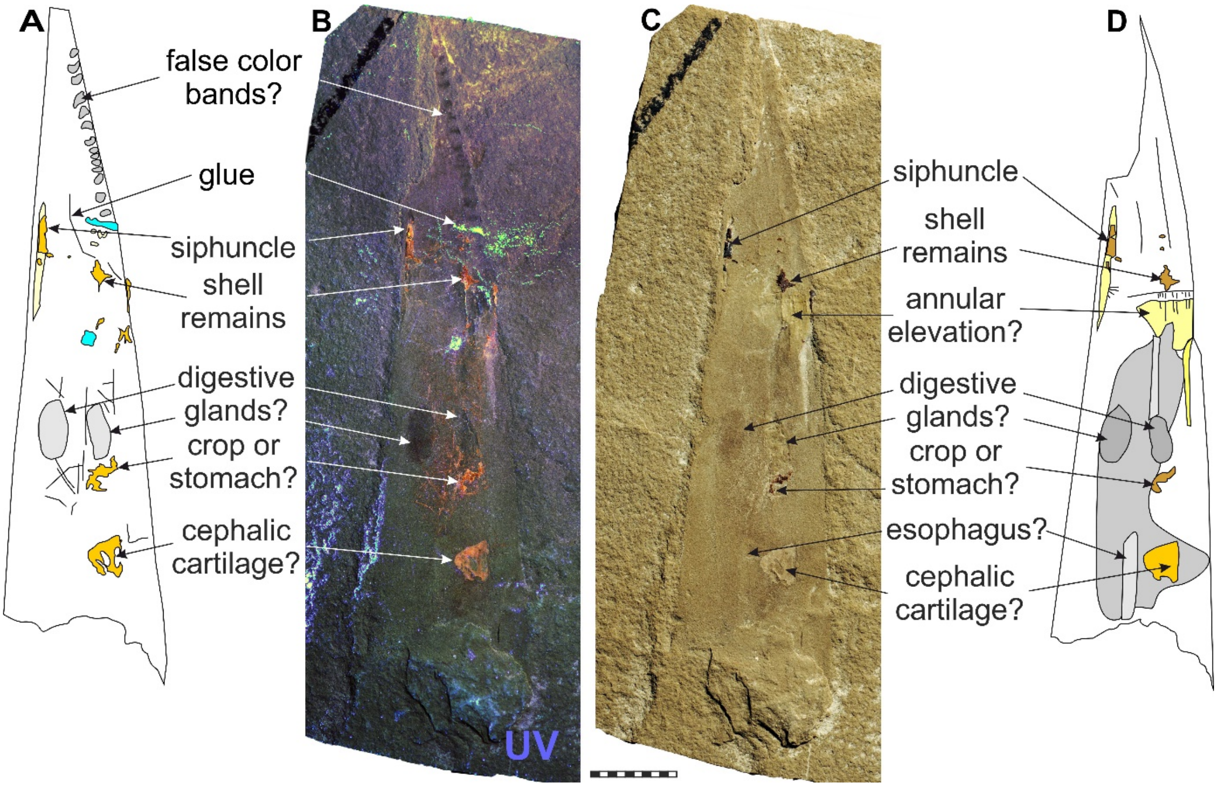

Supplementary Figure 3. *Gordoniconus beargulchensis*, CM 52640. Scale bar is 10 mm. This specimen shows the siphuncle, possible false color patterns and possibly paired oval spots; if really paired they might be digestive glands, if unpaired it might be an ink sac in an untypical preservation. A, sketch of B. B, UV-image. C, photo taken under white light. D, sketch of C.

Supplementary RTI- and PTM-files. RTI-imagery (Reflectance Transformation Imaging) of the holotype of *Gordoniconus beargulchensis* (AMNH 50267). To view it, please download RTIViewer available at http://culturalheritageimaging.org/What_We_Offer/Downloads/View/

Files can be downloaded using the following links:

RTI-file : RTI_Gordoniconus_AMNH43264v2.rti, doi 10.6084/m9.figshare.8319989

PTM-file: RTI_Gordoniconus_AMNH43264v1.ptm, doi 10.6084/m9.figshare.8319983
