# Supplementary material for: Anatomy and evolution of the first Coleoidea in the Carboniferous
Source: Commun Biol. 2019 Jul 31;2:280. doi: 10.1038/s42003-019-0523-2 (PMC6668408; doi:10.1038/s42003-019-0523-2)
Supplement: Supplementary file 3 — Description of additional supplementary items [file 42003_2019_523_MOESM3_ESM.docx]

**Description of Additional Supplementary Files**

**File Name**: Supplementary Data

**Description**:  RTI image file
