# Supplementary material for: Anatomy and evolution of the first Coleoidea in the Carboniferous
Source: Commun Biol. 2019 Jul 31;2:280. doi: 10.1038/s42003-019-0523-2 (PMC6668408; doi:10.1038/s42003-019-0523-2)
Supplement: Supplementary file 4 — Reporting Summary [file 42003_2019_523_MOESM4_ESM.pdf]

nature research

Corresponding author(s): Christian Klug

Last updated by author(s): Jun 17, 2019

Reporting Summary

Nature Research wishes to improve the reproducibility of the work that we publish. This form provides structure for consistency and transparency in reporting. For further information on Nature Research policies, see Authors & Referees and the Editorial Policy Checklist.

Statistics

For all statistical analyses, confirm that the following items are present in the figure legend, table legend, main text, or Methods section.

n/a	Confirmed	
☐	☒	The exact sample size (*n*) for each experimental group/condition, given as a discrete number and unit of measurement
☐	☒	A statement on whether measurements were taken from distinct samples or whether the same sample was measured repeatedly
☒	☐	The statistical test(s) used AND whether they are one- or two-sided<br>*Only common tests should be described solely by name; describe more complex techniques in the Methods section.*
☒	☐	A description of all covariates tested
☒	☐	A description of any assumptions or corrections, such as tests of normality and adjustment for multiple comparisons
☒	☐	A full description of the statistical parameters including central tendency (e.g. means) or other basic estimates (e.g. regression coefficient) AND variation (e.g. standard deviation) or associated estimates of uncertainty (e.g. confidence intervals)
☒	☐	For null hypothesis testing, the test statistic (e.g. *F*, *t*, *r*) with confidence intervals, effect sizes, degrees of freedom and *P* value noted<br>*Give P values as exact values whenever suitable.*
☒	☐	For Bayesian analysis, information on the choice of priors and Markov chain Monte Carlo settings
☒	☐	For hierarchical and complex designs, identification of the appropriate level for tests and full reporting of outcomes
☒	☐	Estimates of effect sizes (e.g. Cohen's *d*, Pearson's *r*), indicating how they were calculated

Our web collection on statistics for biologists contains articles on many of the points above.

Software and code

Policy information about availability of computer code

Data collection	does not apply
Data analysis	does not apply

For manuscripts utilizing custom algorithms or software that are central to the research but not yet described in published literature, software must be made available to editors/reviewers. We strongly encourage code deposition in a community repository (e.g. GitHub). See the Nature Research guidelines for submitting code & software for further information.

Data

Policy information about availability of data

All manuscripts must include a data availability statement. This statement should provide the following information, where applicable:

- Accession codes, unique identifiers, or web links for publicly available datasets
- A list of figures that have associated raw data
- A description of any restrictions on data availability

RTI-file is available online.

Field-specific reporting

Please select the one below that is the best fit for your research. If you are not sure, read the appropriate sections before making your selection.

☐ Life sciences ☐ Behavioural & social sciences ☒ Ecological, evolutionary & environmental sciences

For a reference copy of the document with all sections, see nature.com/documents/nr-reporting-summary-flat.pdf

Ecological, evolutionary & environmental sciences study design

All studies must disclose on these points even when the disclosure is negative.

Study description	We examined fossil cephalopods using various light sources.
Research sample	Fossil specimens kept at the AMNH in New York, Carnegie Museum of Natural History, Pittsburgh, Pennsylvania (CM) and the University of Montana Paleontology Center Research Collection, Missoula, Montana (UMPC).
Sampling strategy	We looked at as many specimens showing soft-tissue remains as possible and selected those showing most detail in the body chamber. The holotype is the best specimen and thus, we focused mainly on this specimen.
Data collection	Photographs were taken under white light, UV-light and various other wave lengths. Additionally, µXRF mapping was performed at the DiffAbs beamline of the SOLEIL synchrotron source (France) using a monochromatic beam of 18.2 keV, selected for excitation of K-lines from phosphorus to zirconium and L-lines from cadmium to uranium.
Timing and spatial scale	Does not apply.
Data exclusions	Does not apply.
Reproducibility	All specimens can be studied at the institutions listed above. When the same light sources are chosen, similar imagery can be produced easily. The results can then be compared with our interpretations.
Randomization	Does not apply.
Blinding	Does not apply.

Did the study involve field work? ☐ Yes ☒ No

Reporting for specific materials, systems and methods

We require information from authors about some types of materials, experimental systems and methods used in many studies. Here, indicate whether each material, system or method listed is relevant to your study. If you are not sure if a list item applies to your research, read the appropriate section before selecting a response.

Materials & experimental systems

n/a	Involved in the study	
☒	☐	Antibodies
☒	☐	Eukaryotic cell lines
☐	☒	Palaeontology
☒	☐	Animals and other organisms
☒	☐	Human research participants
☒	☐	Clinical data

Methods

n/a	Involved in the study	
☒	☐	ChIP-seq
☒	☐	Flow cytometry
☒	☐	MRI-based neuroimaging

Palaeontology

Specimen provenance	Bear Gulch, Montana
Specimen deposition	All fossil specimens kept at the AMNH in New York, Carnegie Museum of Natural History, Pittsburgh, Pennsylvania (CM) and the University of Montana Paleontology Center Research Collection, Missoula, Montana (UMPC).
Dating methods	No new datations were carried out since the locality is quite well studied and the age is readily known.

☒ Tick this box to confirm that the raw and calibrated dates are available in the paper or in Supplementary Information.
